# Antidepressant-like effect of dehydrozingerone from *Zingiber officinale* by elevating monoamines in brain: in silico and in vivo studies

**DOI:** 10.1007/s43440-021-00252-0

**Published:** 2021-06-28

**Authors:** Sudheer Moorkoth, N. Sai Prathyusha, Suman Manandhar, Yuanxin Xue, Runali Sankhe, K. S. R. Pai, Nitesh Kumar

**Affiliations:** 1 Department of Pharmaceutical Quality Assurance, Manipal College of Pharmaceutical Sciences, Manipal Academy of Higher Education, Manipal, Karnataka 576104 India; 2 Department of Pharmacology, Manipal College of Pharmaceutical Sciences, Manipal Academy of Higher Education, Manipal, Karnataka 576104 India; 3 Faculty of Health Sciences, McMaster University, Hamilton, Ontario Canada; 4 Department of Pharmacology and Toxicology, National Institute of Pharmaceutical Education and Research, Hajipur, Bihar 844102 India

**Keywords:** Dehydrozingerone, Depression, Monoamine oxidase-A, Neurotransmitter, Molecular dynamics, LCMS

## Abstract

**Background:**

Dehydrozingerone (DHZ) is an active ingredient of *Zingiber officinale* and structural half analogue of curcumin. In the present study, DHZ was evaluated for monoamine oxidase (MAO) inhibitory activity in silico and antidepressant activity in vivo.

**Method:**

The binding affinity of DHZ with MAO-A (PDB ID: 2Z5Y) was assessed using Schrodinger's Maestro followed by free energy calculation, pharmacokinetic property prediction using Qikprop and Molecular dynamics simulation using Desmond. In vivo antidepressant activity of DHZ was evaluated on C57 BL/6 male mice using Escilatopram as the standard antidepressant. Open field test (OFT), forced swimming test (FST) and tail suspension test (TST) were used to evaluate the antidepressant effect of the drugs on days 1 and 7. Following the behavioural study, neurotransmitters (noradrenaline, dopamine and serotonin) were estimated using liquid chromatography–mass spectrometry.

**Results:**

DHZ demonstrated a greater binding affinity for the MAO-A enzyme compared to moclobemide in silico. Immobility in TST and FST were significantly (*p* < 0.05) reduced in vivo with 100mg/kg DHZ as compared to respective controls. DHZ treatment was more effective 1 h post treatment compared to vehicle control. A significant increase in levels of neurotransmitters was observed in mice brain homogenate in response to DHZ treatment, reassuring its antidepressant-like potential.

**Conclusion:**

DHZ demonstrated MAO-A inhibition in silico, and the increased neurotransmitter levels in the brain in vivo were associated with an antidepressant-like effect.

## Introduction

Depression is the most prevalent mental disorder worldwide affecting more than 300 million people of all ages. The chronic and severe form of depression often leads to suicidal tendencies, with approximately 800,000 people committing suicide each year, primarily within the age group of 15–29 years old. The serendipitous discovery of the first monoamine oxidase (MAO) inhibitor occurred after observing the side effects of an antitubercular drug in the 1950s, Iproniazid, which induced euphoria, sleep improvement and psychostimulation [1]. The role of several neurotransmitters such as serotonin, norepinephrine/epinephrine and dopamine has been indicated in depression [2, 3]. Currently, available antidepressants drugs (tricyclic and tetracyclic antidepressants, MAO inhibitors, selective serotonin and noradrenaline reuptake inhibitors and mood stabilizers) are prescribed based on the medical condition and preferences [4]. The use of tricyclic antidepressants and monoamine oxidase inhibitors have shown short term reversal of the symptoms of depression. Currently available antidepressants have low efficacy and dietary restrictions, and some patients are non-responsive altogether [5]. There also exist numerous side effects including liver toxicity and hypertension [6].

MAO, a flavin adenine dinucleotide present in the mitochondrial outer membrane, is involved in the oxidative deamination of monoamines including serotonin, dopamine, epinephrine, norepinephrine, melatonin, etc. MAO has two subtypes, MAO-A and MAO-B, whose amino acid sequences are up to 70% identical, although each enzyme has a unique substrate and inhibitor specificities [7]. MAO inhibitors are clinically used as therapeutics for depression, Parkinson's and Alzheimer's disease. It stops the breakdown of these monoamine neurotransmitters resulting in an increased concentration and availability [8].

MAO inhibitors are one of the important therapeutic options for depression. However, due to the adverse effect and serious drug and food interactions with the MAO inhibitor, their use has been reduced. Natural compounds have great potential for drug design and discovery for several disease conditions without the risk of several adverse effects. Dehydrozingerone (DHZ) (4-hydroxy-3-methoxybenzalacetone), is the phenolic compound extracted from the rhizome of *Zingiber officinale* having potent antioxidant, tyrosinase inhibitory, anti-inflammatory, antidepressant, antifungal and anticancer activities [9, 10]. Ginger is one of the important plants traditionally used since the ancient period for its medicinal property especially in Unani, Ayurveda and Chinese medicinal systems [11]. Traditional Chinese Medicinal decoction prescribed since 1000 years for the treatment of depression, which consists of five indigenous herbs namely, *Pinellia ternata *(*Thunb.*)* Breit, Magnolia officinalis Rehd. et Wils., Poria cocos *(*Schw.*)* Wolf, Perilla frutescens *(L.)* Britt. and Zingiber officinale* Rosc [11–13]*.* Ginger rhizome has been traditionally used as one of the components in the herbal formula Banxia houpu decoction for the treatment of mental disorders including depression [14]. Dehydrozingerone is the phenolic compound isolated from the rhizome of *Zingiber officinale* [9]*.* It is the half-structural analogue of curcumin which can be synthesized by the aldol condensation reaction between vanillin and acetone in the laboratory. DHZ has been reported to possess cytotoxic, [15] antimicrobial activity, antioxidant property, antifungal activity [16], anti-inflammatory activity [17]. Antithrombotic activity of DHZ has been done in the endothelial cells showing its potential as a therapeutic agent in atherosclerosis. Antiprostate cancer property of DHZ has been proven both in vitro as well as in vivo [18]. The antidepressant activity of curcumin has been widely studied and has been linked to the MAO inhibitory activity [19], through the serotonergic and dopaminergic system [20]. Curcumin was found to have potent inhibitory activity for both MAO-A and MAO-B enzymes [21]. DHZ has been reported to have potent antidepressant activity by in vivo study through the involvement of serotonergic and noradrenergic systems [22]. Antidepressant activity of the hydroalcoholic extract from the rhizomes of *Zingiber officinale* has been proven. This study aims to evaluate the involvement of MAO-A inhibitory activity of DHZ for its antidepressant activity by computational based in silico molecular docking and molecular dynamics simulation study followed by in vivo rodent model and LC–MS-based estimation of neurotransmitter level.

## Materials and methods

### In silico docking study

The computational study and analysis were carried out using Maestro 2018–3 molecular modelling platform by Schrodinger on a Linux operating system, Intel core processor with 8 GB RAM, utilizing tools like Ligprep, Protein preparation wizard, GLIDE (Grid-based Ligand Docking with Energetic), Prime, Desmond and Qikprop.

#### Ligand and protein preparation

The 2D SDF format of DHZ and standard antidepressant moclobemide (MAO-A inhibitor) was obtained from the ZINC database. DHZ was prepared for docking analysis using the ‘LigPrep’ tool wherein the conversion to 3D form and generation of possible tautomers and conformers were completed. Ligand preparation was completed at neutral pH 7 ± 2 using Epik, and energy minimization of the ligand was under OPLS3e (Optimized potentials for liquid simulations_3e) force field [23].

The inhibitor bound structure of MAO-A (PDB ID: 2Z5Y) with a resolution of 2.17 Å was retrieved from RCSB Protein Data Bank [7] and prepared using the ‘Protein preparation Wizard’ tool. It involves three steps including viz. import and processing; review and modify; and refinement of protein. Initially, the protein was pre-processed by the addition of hydrogen atoms, removal of unwanted water molecules beyond 5 Å from the hetero group and addition of missing side chains and loops. The pre-processed protein was then refined by hydrogen bond assignment at neutral pH using PROPKA and energy minimization using OPLS3e force field.

#### Molecular docking

The grid was generated in the protein using the receptor grid generation tool of the GLIDE module, considering the protein-bound inhibitor. GLIDE involves a rapid, accurate and systematic search for the conformational, positional and orientational space followed by docking and scoring [24]. The prepared ligands were then docked to the active site of MAO-A, using the 'Ligand docking' tool in the GLIDE module with the XP (Extra precision) mode of docking, known as rigid docking. Only the hydrogen bonds (H-bond) formed between acceptor–donor atoms with a distance less than 3.3 Å were considered for this study. Computationally, XP docking is the more extensive mode of docking which uses the advanced scoring function. In addition, chances of getting false-positive results are less compared to high-throughput virtual screening and standard precision docking mode.

The validation of the generated grid was done by the re-docking of the known co-crystallized ligands present in the same protein (PDB ID: 2Z5Y). RMSD value was measured to check the suitability of the generated grid and docking results [25].

#### Drug-likeness evaluation

The 'QikProp’ module was used to study the absorption, distribution, metabolism and excretion/toxicity properties of DHZ. The physiochemical properties including molecular weight (Mol wt), QPlogS (solubility), QPlog HERG (ability to block K^+^ channel), QPPCaco (Caco2 cell permeability), QPlogBBB (Blood/brain partition coefficient), human oral absorption (% oral absorption) and rule of five were evaluated to provide a basic understanding of the pharmacokinetic property of the molecule and predict the drug-likeness of the molecule.

#### Molecular dynamics (MD) simulation

Drugs and proteins are surrounded by water molecules inside the body and are continuously in motion. MD simulation helps us understand a similar situation by mimicking physiological condition. MD simulation study further confirms the binding of the ligands and the stability of the complex in simulated physiological conditions. For a better understanding of the binding affinity and stability of DHZ with MAO-A, MD simulation study was performed using the ‘Desmond’ module. Three steps are involved in MD simulation, (1) System builder: The protein–ligand complex system was built using simple point charged water model (SPC) in the orthorhombic water box of dimension 10 Å × 10 Å × 10 Å and the system was neutralized either using Na^+^ or Cl^−^ ions as per the requirement. NPT (Normal Pressure and Temperature) simulation system was used, and the temperature and pressure of the system were maintained at 300 K and 1 bar, respectively, during the simulation study. (2) Minimization: Before running the simulation, the receptor–ligand complex in the water box was minimized. (3) Molecular dynamics: the trajectory of the simulation was recorded every 30 ps, and approximately 1000 frames were recorded for 30 ns simulation period. The root mean square deviation (RMSD) was calculated for DHZ.

### In vivo study

#### Experimental animals

C57BL/6 male mice of 4 months age weighing 25–30 g were obtained from the Central Animal Research Facility (CARF) of Manipal Academy of Higher Education, Manipal (Karnataka, India). Animals were acclimatized for one week in a controlled condition of temperature (25 ± 2 °C) with 12 h of light and 12 h of dark cycles in laboratory conditions. Before two hours of drug administration, the animals were kept fasting to increase drug absorption. Prior approval was obtained from Institutional Animals Ethics Committee (IAEC), Kasturba Medical College, Manipal Academy of Higher Education; IAEC number: Reg No.94/PO/ReBi/S/99/CPCSEA Dated 28/04/2017 and the experimental procedures were done as per the CPCSEA guidelines.

#### Drugs and chemicals

Noradrenaline (Sigma Aldrich > 98% purity), Serotonin hydrochloride (Sigma Aldrich > 98% purity) and Dopamine hydrochloride (Sigma Aldrich > 98.5% purity) were procured from Durga Laboratories, Mangalore. Acetonitrile (LC–MS grade Biosolve) was purchased from Ultra International Lab, Bangalore. Formic acid, ethanol and sodium carboxymethylcellulose were obtained from HiMedia Laboratories Pvt. Ltd., Mumbai, India.

#### Synthesis of DHZ

DHZ was synthesized using the method as described by Agrawal et al., [26] with a slight modification to increase the yield. 2.5 g of vanillin and 20 ml of acetone were stirred with a magnetic stirrer for 25 min. 15 ml sodium hydroxide solution (0.25 M) was added and stirred continuously for another two hours. Ice was added to this solution followed by the addition of 10% HCl with continuous stirring until a yellow precipitate formed. The precipitate was then filtered and dried for 24 h. Recrystallization was done with hot ethanol. The final product was characterised by mass and infrared spectroscopy.

#### Treatment schedule

Animals were allocated to five groups (n = 6 per group), namely normal/vehicle control, escitalopram (10 mg/kg, *po*) and DHZ (100 mg/kg *po*) for three time points (1 h, 3 h and 6 h groups). Dose for escitalopram and DHZ were based on the previous reports [26, 27]. All doses were freshly prepared in 0.25% CMC and animals were given treatment for seven days. Behavioural tests were conducted on the first and seventh day after 1 h, 3 h and 6 h of dosing. The behavioural studies were performed consecutively in the following order with a gap of 1 min between each test: open field test (OFT), forced swim test (FST) and tail suspension test (TST).

#### Behavioural tests

##### Forced swim test (FST)

After 1 h, 3 h and 6 h following the treatment of DHZ, vehicle or escitalopram, mice were placed in the cylindrical glass tank (height: 80 cm, diameter: 30 cm) filled with water up to a height of 40 cm [28]. The mice were not allowed to touch the bottom or jump out of the cylinder during the test. The duration of immobility (floating with only small movements, keeping the head above the water surface) and active swimming shown by the mice were noted during the 6-min test session.

##### Open field test (OFT)

The mice were placed in the OFT device (glass chamber consisting of 9 squares, 30 cm × 30 cm each) and observed 1 h, 3 h and 6 h post DHZ and escitalopram administration for a total period of 8 min (1 min acclimatization and 7 min testing). The arrangement of light was set to a minimum to avoid the anxiety behaviour of the mice. The apparatus was cleaned with 70% ethanol each time animals were removed. The mice were placed in the left corner at the start of the trial and were observed for the number of line crossing and centre square entry. Scoring was made for these behavioural parameters during the entire test. OFT is done to determine the exploratory and depressive behaviour of the rodents [22, 29].

##### Tail suspension test (TST)

The tail suspension test was done as per the method suggested by Steru et al [30]. Mice were suspended 50 cm above the floor at the edge of a table after 1 h, 3 h and 6 h of the last day of treatment with DHZ and escitalopram. The adhesive tape was placed approximately 1 cm from the tail tip. The immobility time or absence of escape-oriented behaviour was noted during the 6-min testing period [31, 32].

#### Estimation of neurotransmitter

##### Isolation of brain and Preparation of sample

After completion of the behavioural studies, mice were sacrificed, the brain was isolated and stored at − 80 °C. Brain samples were weighed and homogenized using ice-cold 2% formic acid in water (15 ml/g), then centrifuged for 60 min at 12,000 RPM at 4 °C [33]. The supernatant was processed, and internal standards (ISTD, Isoprenaline) were added. In order to extract noradrenaline, dopamine and serotonin from the matrix, formic acid with acetonitrile and water were added to homogenize the brain, which was then centrifuged again for 10 min at 12,000 RPM. Then, the supernatant was evaporated to dryness at 45 °C for 1 h using a nitrogen evaporator [34]. Finally, the residue was dried and reconstituted with 200 µl of the mobile phase.

##### LC–MS method optimization

A Thermo Scientific (MA, USA) UHPLC–MS with Dionex Ultimate 3000 liquid chromatograph interfaced with a linear ion-trap analyzer and an electron spray ionization source was used. MS and chromatographic method development were performed using LTQ XL (MA, USA) and Chromeleon (MA, USA) softwares, respectively. Batch analysis was done using the Xcalibur software (MA, USA) and quantification was done using LC Quan software (MA, USA).

### Statistical analysis

The data obtained were analyzed using Graph Pad Prism using one-way or two-way ANOVA, followed by Tukey’s post hoc test. All the results were expressed as mean ± SEM. *p* < 0.05 was considered statistically significant at 95% confidence interval.

## Results

### In silico* results*

#### Prediction of drug-likeness

Physicochemical descriptors and properties such as molecular weight, predicted central nervous system (CNS) activity, QPlogo/w, QPlogHERG, QPlogBBB, QPlogCaco, QPlogS, and % human oral absorption were estimated using ‘QikProp' tool in Maestro. DHZ demonstrated a predicted percentage of human oral absorption of 89.89%. Predicted apparent QPPCaco for DHZ was found to be 949.609 nm/sec. This indicates better intestinal absorption property of the DHZ molecule. All the pharmacokinetic properties for DHZ showed optimum values for QPlogS, QPlogo/w and QPlogHERG, predicting the drug-likeness of DHZ. The results are presented in Table 1.

**Table 1:** Predicted physicochemical properties of DHZ

Title	Mol Wt	CNS	QPlogPo/w	QplogS (mol dm–3)	QPPCaco	QPlogHERG	QplogBB	Rule of 5	% Human oral absorption
Recommended values	130.0–725.0	− 2.0 to 2.0	− 2.0 to 6.5	− 6.5 to 0.5	< 25 poor, > 500 great	Below − 5	− 3.0–1.2	Max-4	> 80%: high < 25%: low
Observed values for DHZ	192.214	0	1.650	− 2.321	949.609	− 4.288	− 0.688	0	89.899

#### Molecular docking analysis

The molecular docking predicts binding orientation and structural stability of the ligand–protein complex. The docking process involves the search algorithm in the generation of different poses and scoring function that is involved in the evaluation of the binding interaction of different poses and protein [25].

**Fig. 1:**
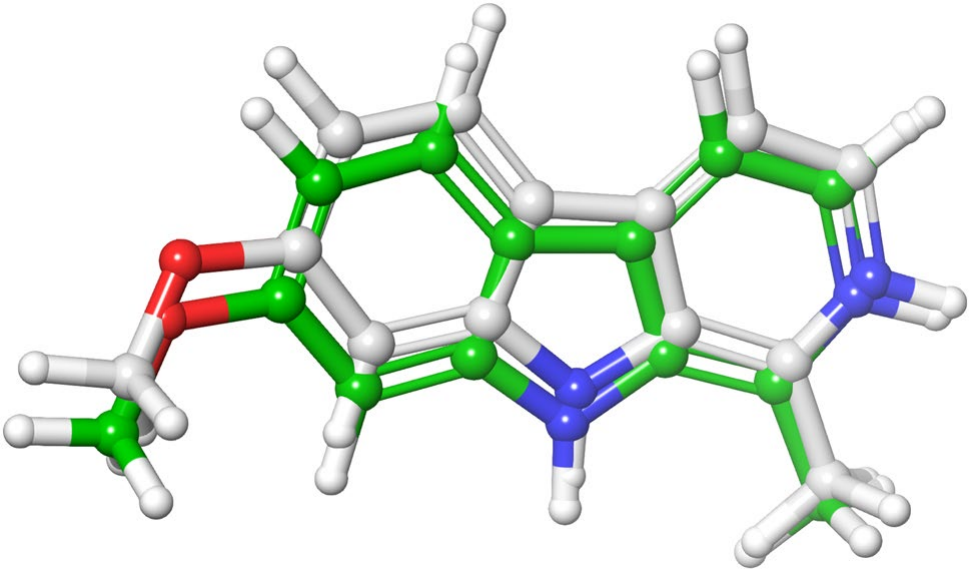
Superposition of the inbound and redocked molecule

The validation of the generated grid was done by redocking the inbound ligand and calculating the RMSD. RMS of 0.5235 Ǻ was observed after the superimposition of redocked ligand and protein inbound ligand, which validates the generated grid, as seen in Fig. 1. XP docking study was performed which suggested that the docking score of DHZ was better than the standard mooclobemide molecule, as listed in Table 2.

**Table 2:**
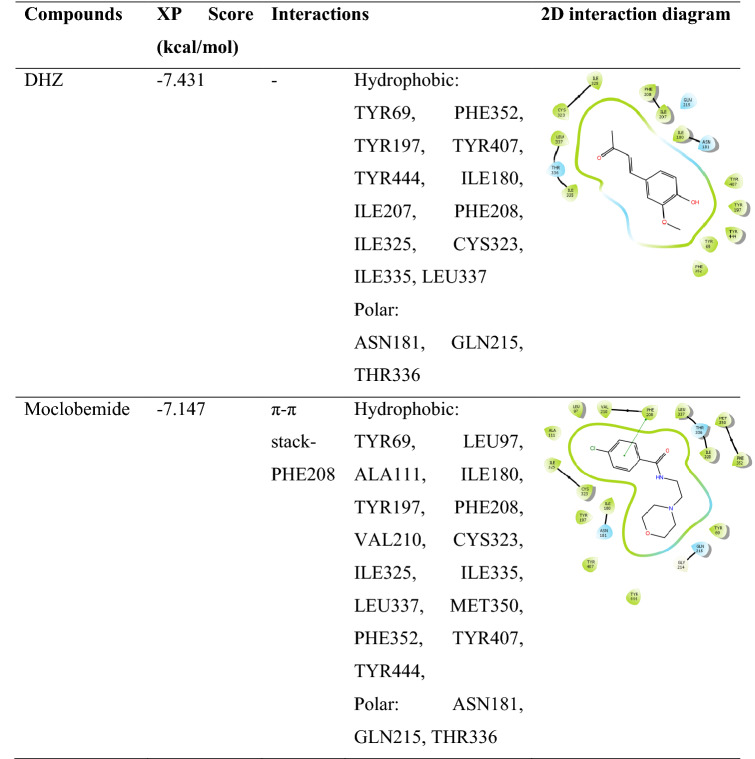
Binding and nonbonding interactions with the XP score of DHZ and Moclobemide

The inhibitor binding site for MAO-A consists of the cavity with a flavin molecule extending from residues 210–216 [35]. The natural ligands inhibiting MAO-A has been reported to show π–π interactions with TYR444, PHE208, TYR407 and PHE352 as well as H-bond interactions with ASN 181, TYR197, and TYR444 [36]. DHZ with XP score of -7.431 showed hydrophobic interactions with TYR69, PHE352, TYR197, TYR407, TYR444, ILE180, ILE207, PHE208, ILE325, CYS323, ILE335, and LEU337 and polar interactions with ASN181, GLN215, and THR336. A similar interaction was observed in moclobemide with XP score of − 7.147 and π–π stack interaction with PHE208.

#### Molecular dynamics (MD) simulation

**Fig. 2:**
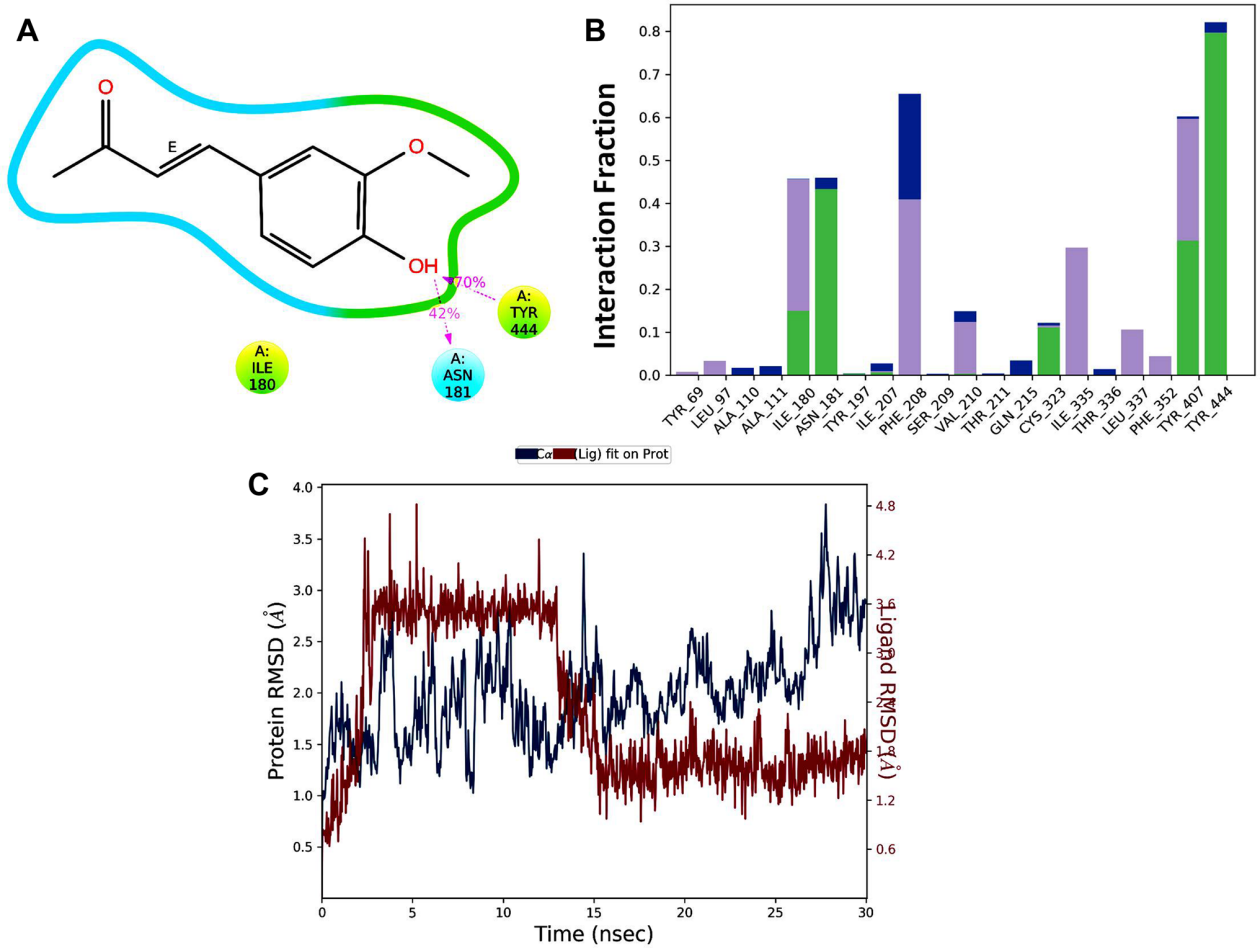
Molecular dynamics simulation of MAO-A with DHZ. **a** 2D interaction diagram of DHZ during MD simulation. **b** Plot showing the interaction of MAO-A and DHZ molecule throughout the simulation. **c** RMSD plot of DHZ and MAO-A protein complex

Molecular docking can only predict the interaction of the static protein and ligand. To understand the interaction pattern of the ligands with the dynamic protein in an environment similar to physiological conditions, DHZ was subjected to the MD simulation study for 30 ns. A stable RMSD of 3.6 Å was observed for DHZ for 3–12 ns duration. The formation of the H-bond and water bridge interactions between the hydroxyl group of DHZ and residue ASN181 was predominant during the 3–12 ns duration. After 12 ns, the H-bond and water bridge type of interactions of ASN181 were lost. Stable interactions, mainly hydrophobic and water bridge, were observed with residue PHE208 of MAO-A and DHZ after 15 ns of the simulation, which remained stable during 30 ns of MD simulation. These interactions have led to a decrease in RMSD of ligand to 1.8 Å. During the XP docking study, there was no H-bond interaction with ILE180, ASN181, CYS323, TYR407 and TYR444 between DHZ and MAO-A, whereas the formation of H-bond interaction was observed during the MD simulation study. For the entire simulation study, residues TYR 407 (mainly H-bond and hydrophobic interaction), TYR444 (mainly H-bond), ILE 180 (mainly H-bond and hydrophobic interaction) and ILE 335 (hydrophobic interaction) were involved in the interaction with the ligand DHZ. The protein molecule was stable throughout the simulation study. RMSD fluctuation from 1.0 to 3.5 Å was observed during the entire simulation study. RMSF plot showing the fluctuation of 4.8 Å in the protein structure was mainly due to the tail region residue from 450 to 500. However, RMSF fluctuation was comparatively less in the case of ligand (1–2 Å), DHZ. MD simulated interactions are represented in Fig. 2.

### In vivo study

#### Characterization of dehydrozingerone

**Fig. 3:**
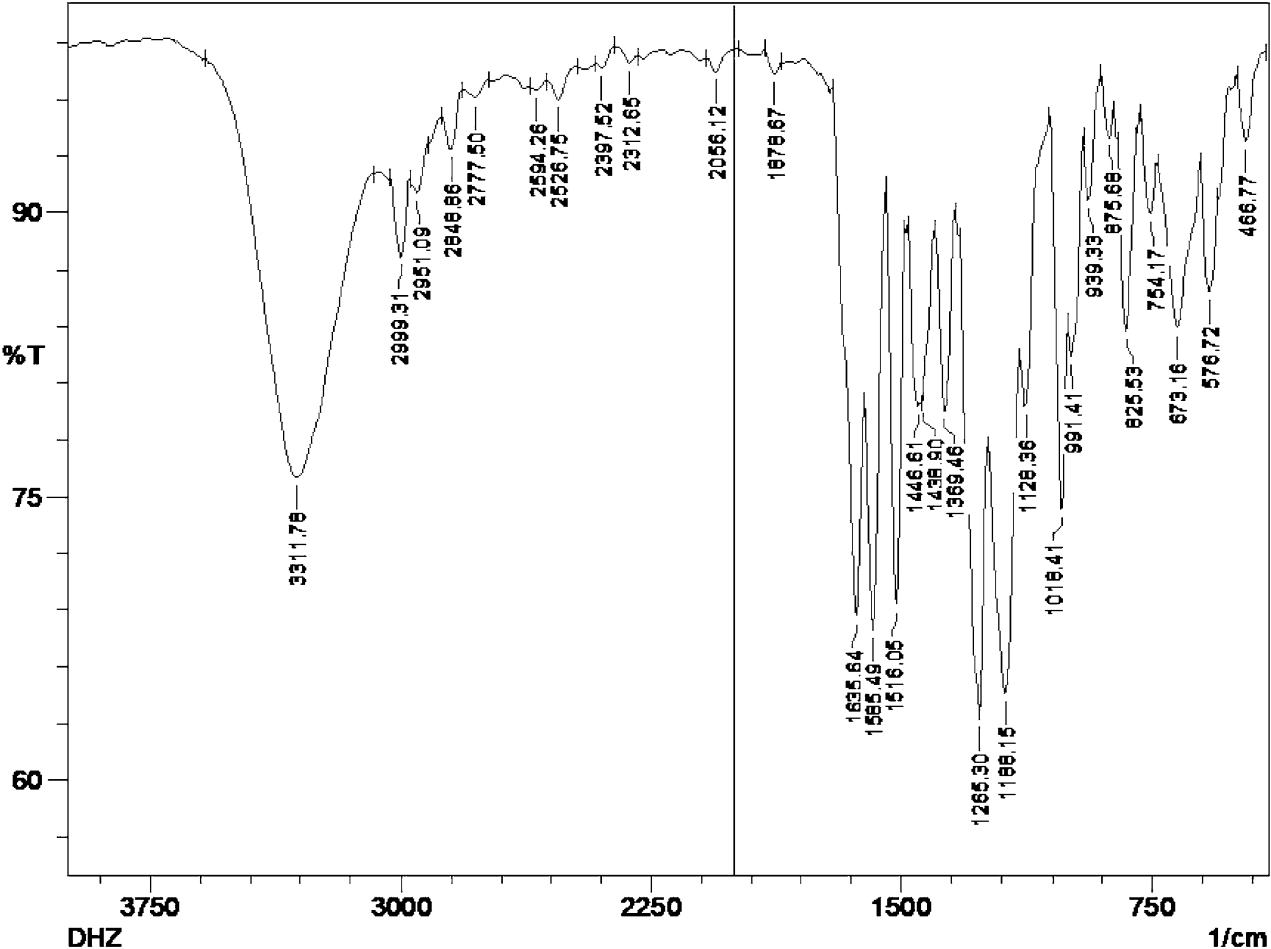
IR data of DHZ

**Fig. 4:**
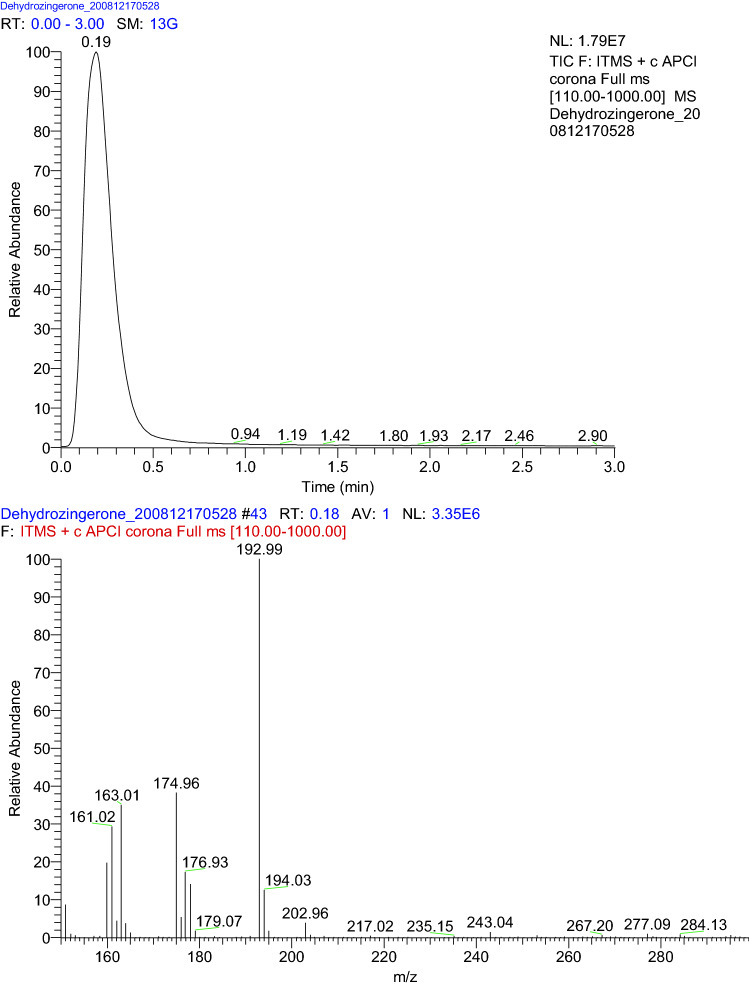
Mass data of DHZ

The FTIR spectra of DHZ showed absorption bands at 3311.78 cm^−1^ due to the OH stretching (-C6H5OH), 2999.31 cm^−1^ (aromatic CH stretch), 2848.86 cm^−1^ (aliphatic CH stretch -OCH3), 1635 carbonyl stretching (–C=C–), 1585.49 cm^−1^ (alkene stretch –C=C–) and 1516 cm^−1^ (aromatic –C=C– stretch) (Fig. 3). The ESI mass spectra in the positive ionization mode illustrated the base peak at 193 m/z, corresponding to the molecular weight of DHZ. (Fig. 4).

#### Effect of DHZ treatment in OFT

*Number of Line Crossings* The number of line crossings in the normal control group was found to be 6.75 ± 1.32 on day 1 and 4.25 ± 0.48 on day 7. The treatment with escitalopram showed a significant increase in the number of line crossings on day 1 (*F*_4,12_ = 14.37, *p* = 0.0023) and day 7 (*F*_4,13_ = 17.11, *p* = 0.0004) as compared to normal control. DHZ treatment did not show any significant change in the number of line crossings on day 1 after 1 h (*F*_4,12_ = 14.37, *p* = 0.9187), 3 h (*F*_4,12_ = 14.37, *p* = 0.8140) and 6 h (*F*_4,12_ = 14.37, *p* = 0.1320) of DHZ dosing compared to normal control. Statistical values 1st day Line crossing was: *F*_DFn,DFd,_ where DFn is degrees of freedom numerator, and DFd is degrees of freedom denominator and *p* value:: *F*_4,12_ = 14.37 and *p* = 0.0002.

**Fig. 5:**
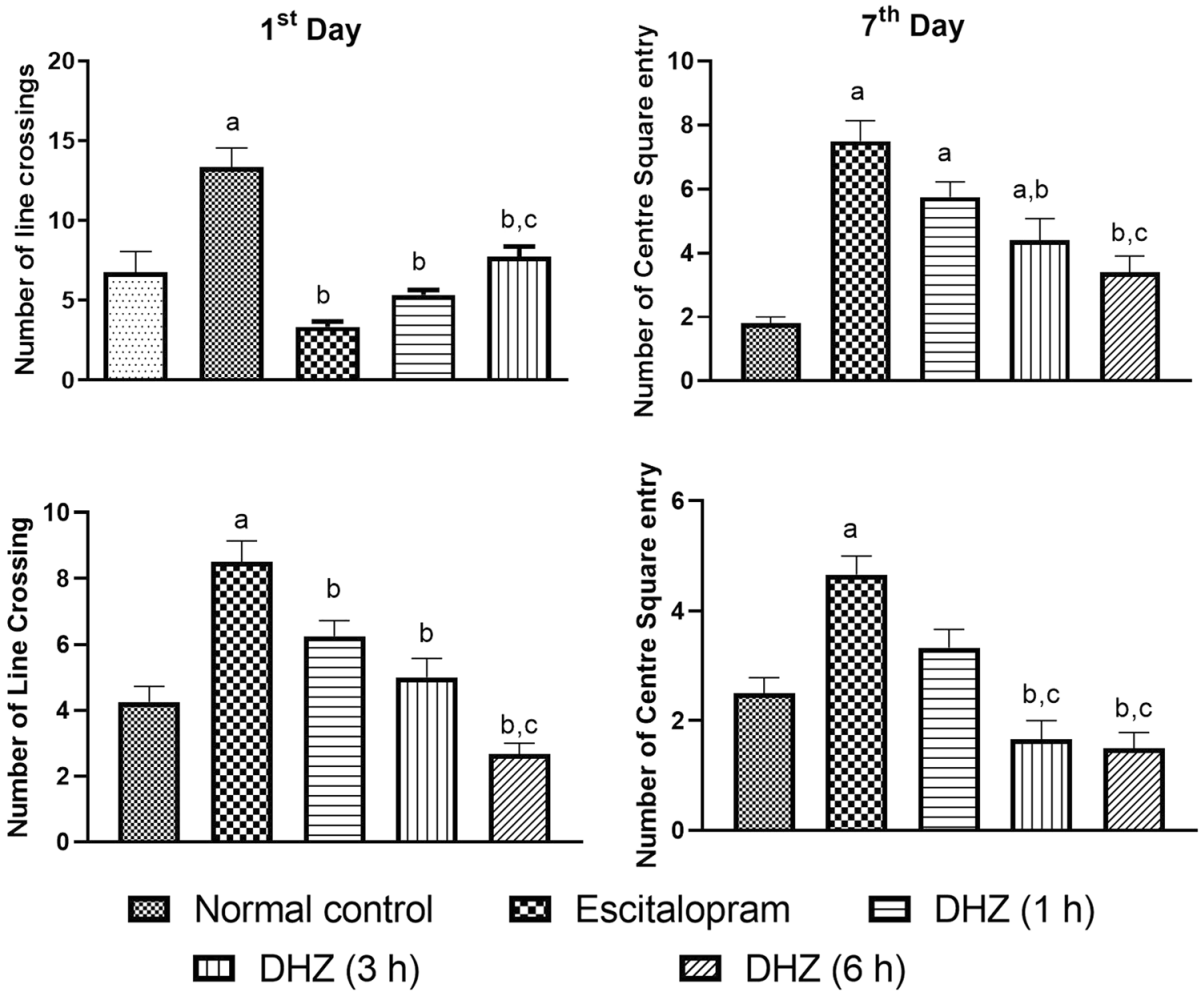
Open Field Test of 1st Day and 7th Day. The results of open field test (OFT) performed on 1st and 7th day of treatment represented as bar diagrams. One-way ANOVA followed by Tukey’s multiple comparison test was used for statistical analysis. The data are presented as mean ± SEM of six animals where ^a^*p* < 0.05 as compared to normal control, ^b^*p* < 0.05 as compared to escitalopram, ^b^*p* < 0.05 as compared to DHZ (1 h), ^c^*p* < 0.05 as compared to DHZ (3 h), ^b^*p* < 0.05 as compared to DHZ (6 h)

Similarly, no significant change in line crossing was seen on day 7 after 1 h (*F*_4,13_ = 17.11, *p* = 0.898), 3 h (*F*_4,13_ = 17.11, *p* = 0.8613) and 6 h (*F*_4,13_ = 17.11, *p* = 0.2929) of DHZ dosing as compared to normal control animals. Statistical values 1st day line crossing were: F_DFn,DFd_ and *p* value:: *F*_4,13_ = 17.11 and *p* < 0.0001. (Fig. 5).

*Number of Centre square entry* The number of centre square entry in the normal control group was found to be 1.80 ± 0.20 on day 1 and 2.50 ± 0.29 on day 7. Treatment with escitalopram showed a significant increase in the number of centre square entry on day 1 (*F*_4,18_ = 16.57, *p* < 0.0001) and on day 7 (*F*_4, 12_ = 16.41, *p* = 0.0027) as compared to normal control animals. DHZ treatment also showed a significant increase in the number of centre square entry on day 1 after 1 h (*F*_4,18_ = 16.57, p = 0.0005) and 3 h (*F*_4,18_ = 16.57, *p* = 0.0141) dosing compared to normal control animals. However, no significant change was observed in this parameter after 6 h of DHZ treatment (*F*_4,18_ = 16.57, *p* = 0.2098) compared to normal control animal on day 1. On day 7, DHZ treatment did not show any significant change in the number of centre square entry after 1 h (*F*_4, 12_ = 16.41, *p* = 0.3723), 3 h (*F*_4, 12_ = 16.41, *p* = 0.3723) and 6 h (*F*_4, 12_ = 16.41, *p* = 0.1675) of dosing as compared to normal control animal. Statistical values were: *F*_DFn,DFd_ and *p* value:: *F*_4,18_ = 16.57 and *p* < 0.0001 on 1st day centre square entry, and *F*4, 12 = 16.41 and *p* < 0.0001 for 7th day centre square entry. (Fig. 5).

#### Effect of DHZ treatment on immobility time of mice in FST and TST

**Fig. 6:**
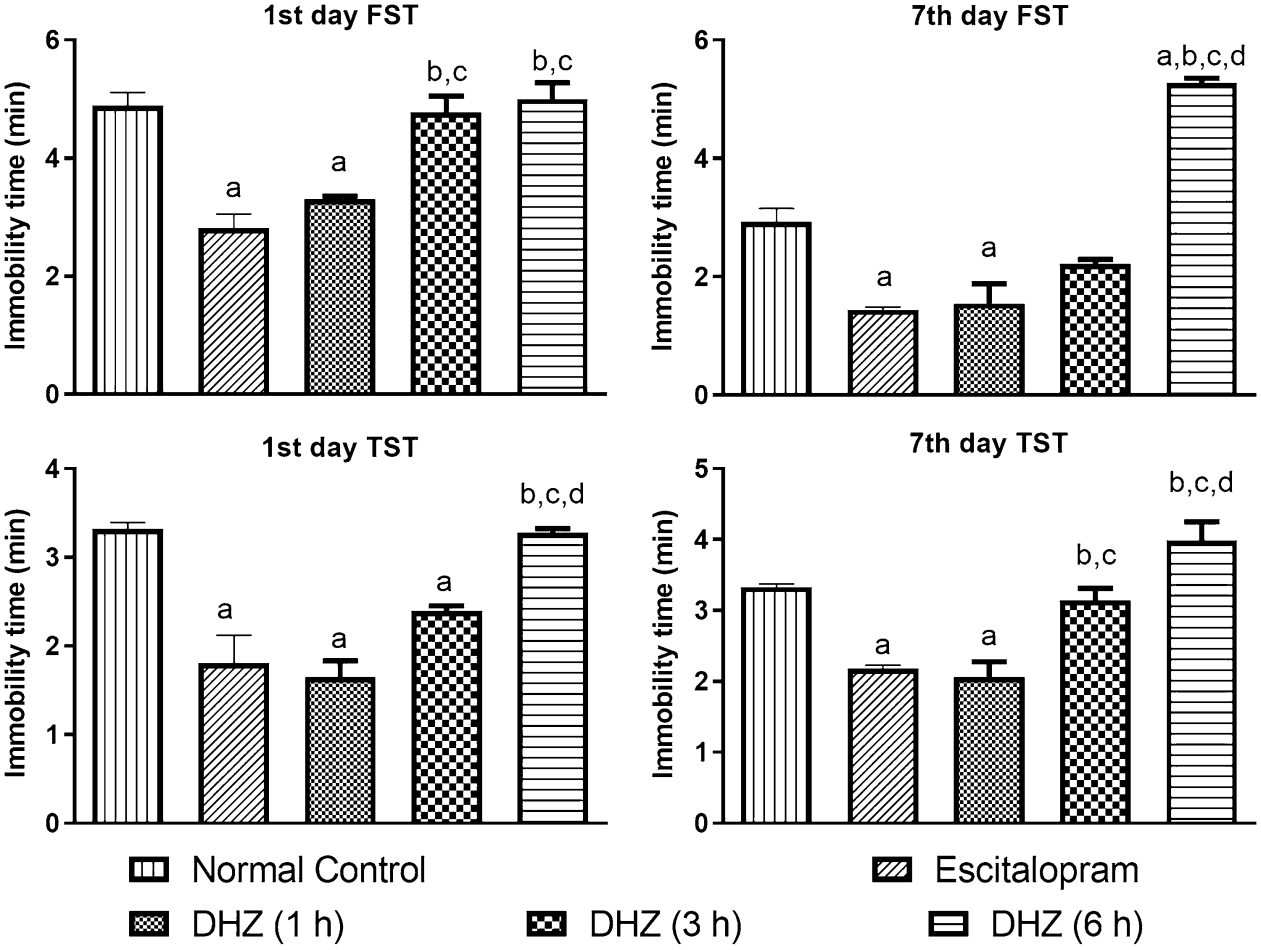
Forced swim test and tail suspension test of 1st day and 7th day. The results of tail suspension test (TST) and forced swim test (FST) performed on 1st and 7th day of treatment represented as bar diagrams. One-way ANOVA followed by Tukey’s multiple comparison test was used for statistical analysis. The data are presented as mean ± SEM of six animals where ^a^*p* < 0.05 as compared to normal control, ^b^*p* < 0.05 as compared to escitalopram, ^b^*p* < 0.05 as compared to DHZ (1 h), ^c^*p* < 0.05 as compared to DHZ (3 h), ^b^*p* < 0.05 as compared to DHZ (6 h)

*FST* The immobility time in the normal control group was 4.89 ± 0.22 s and 2.92 ± 0.52 s on 1st day and 7th day respectively. The immobility time of mice was significantly reduced on both 1st (*F*_4,24_ = 19.53, *p* = 0.0008) and 7th day (*F*_4,18_ = 76.82, *p* = 0004) after 1 h of treatment with DHZ (100 mg/kg *po*) and escitalopram (*F*_4,24_ = 19.53 with *p* < 0.0001 and 1.44 ± 0.05 s with *p* = 0.0002 respectively) as compared to normal control. However, there was no significant reduction in the immobility time in the mice after 3 h (4.77 ± 0.29 s and 2.27 ± 0.07 s) and 6 h of DHZ (5.00 ± 0.27 s and 5.28 ± 0.08 s) treatment on both day 1 and day 7 respectively. Statistical values were as follows: *F*_DFn,DFd_ and *p* value:: *F*_4,24_ = 19.53 and *p* < 0.0001 for FST at day 1; *F*_4,18_ = 76.82 and *p* < 0.0001 for FST at day 7. (Fig. 6).

*TST* The immobility time in normal control animals were 3.32 ± 0.07 s and 3.33 ± 0.05 s on 1st day and 7th day respectively. The immobility time of mice was significantly reduced 1 h post DHZ treatment (*F*_4,22_ = 19.02, *p* < 0.0001 and *F*_4,17_ = 18.68, *p* = 0023) and escitalopram treatment (*F*_4,22_ = 19.02 with *p* < 0.0001 and *F*_4,17_ = 18.68 with *p* = 0.0055) on day 1 and day 7 respectively as compared to normal control. Even 3 h post DHZ treatment showed a significant decrease in immobility (*F*_4,22_ = 19.02 with *p* = 0.0124) on 1st day as compared to normal control animals. However, no significant reduction in the immobility time was observed in the mice 3 h post DHZ treatment (3.14 ± 0.17 s) on day 7 as compared to normal control animals. 6 h post DHZ treatment on day 1 (*F*_4,22_ = 19.02, *p* = 0.9998) and day 7 (*F*_4,17_ = 18.68, *p* = 0.9529) did not show a significant decrease in immobility time as compared to normal control. Statistical values were as follows: *F*_DFn,DFd_ and *p* value:: *F*_4,22_ = 19.02 and *p* < 0.0001 for TST at day 1; *F*_4,17_ = 18.68 and *p* < 0.0001 for TST at day 7. (Fig. 6.)

#### LC–MS method optimization

**Fig. 7:**
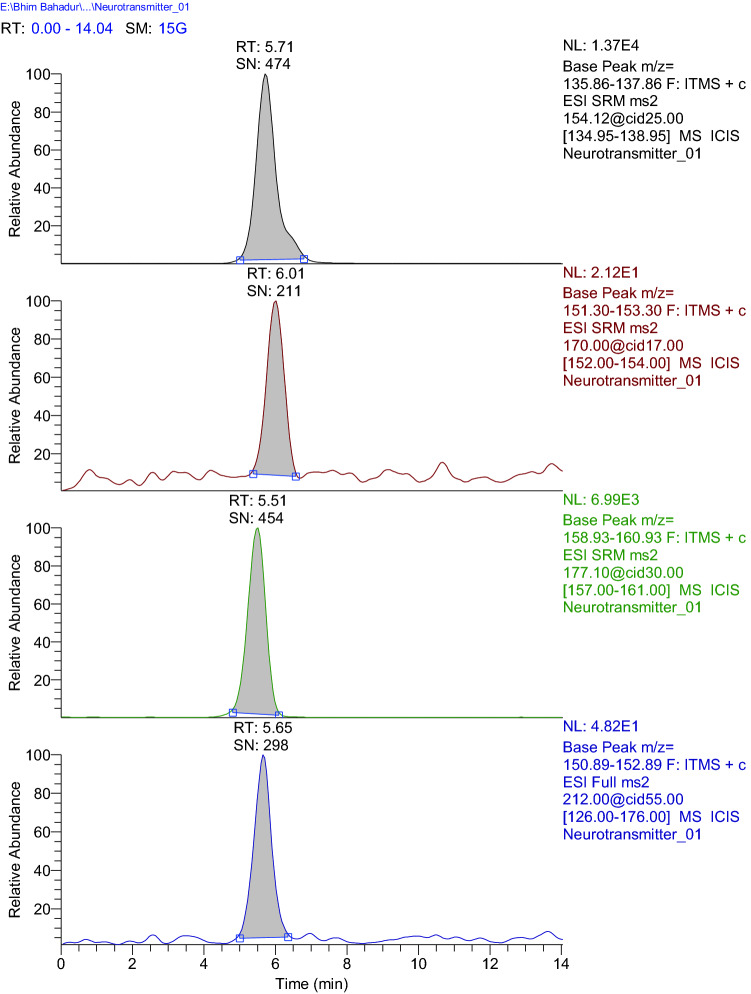
Extracted-ion chromatogram of neurotransmitters. Legend: MS2 extracted ion chromatogram of dopamine (5.71 min), noradrenaline (6.01 min), serotonin (5.51 min) and isoprenaline (Internal standard) (5.65 min)

Optimized chromatographic conditions are as follows: Column: (HILIC Kinetix 50 × 2.1 mm, 2.6 µm), Mobile phase: 0.1% Formic acid in water (A) and 0.1% Formic acid in acetonitrile (B), Gradient programme: (0.0 min %A (80); 2.5 min to 5.0 min %A (70); 9.0 min to 14.0 min %A (80), Flow rate 0.2 mL/min, Injection volume 10 µl; Column temperature: 25 °C; autosampler temperature: 6 °C. The retention time for the analytes in the extracted ion chromatogram in the MS2 mode (Fig. 7) was found to be 5.71, 6.01, 5.51 and 5.65 min respectively for dopamine, noradrenaline, serotonin and isoprenaline (internal standard) [37].

Optimized mass spectrometric conditions are as follows: Ion source: Heated Electron Spray Ionization source (HESI), Analyzer: Ion Trap, Operating mode: Positive polarity with SRM (Selective Reaction Monitoring), Capillary voltage: 48.48 V, Capillary temperature: 300 °C, Source heater temperature: 400 °C, Sheath gas flow rate: 50 arb, Auxiliary gas flow rate: 12 arb, Sweep gas flow rate: 0 arb. SRM transition and collision energy (CE) were as follows: dopamine (154.12–136.86, CE 25), noradrenaline (170.00–152.30, CE 17), serotonin (177.10–159.93, CE 30), isoprenaline (212.00–151.89, CE 55).

#### Estimation of neurotransmitters level in the mice brain

**Fig. 8:**
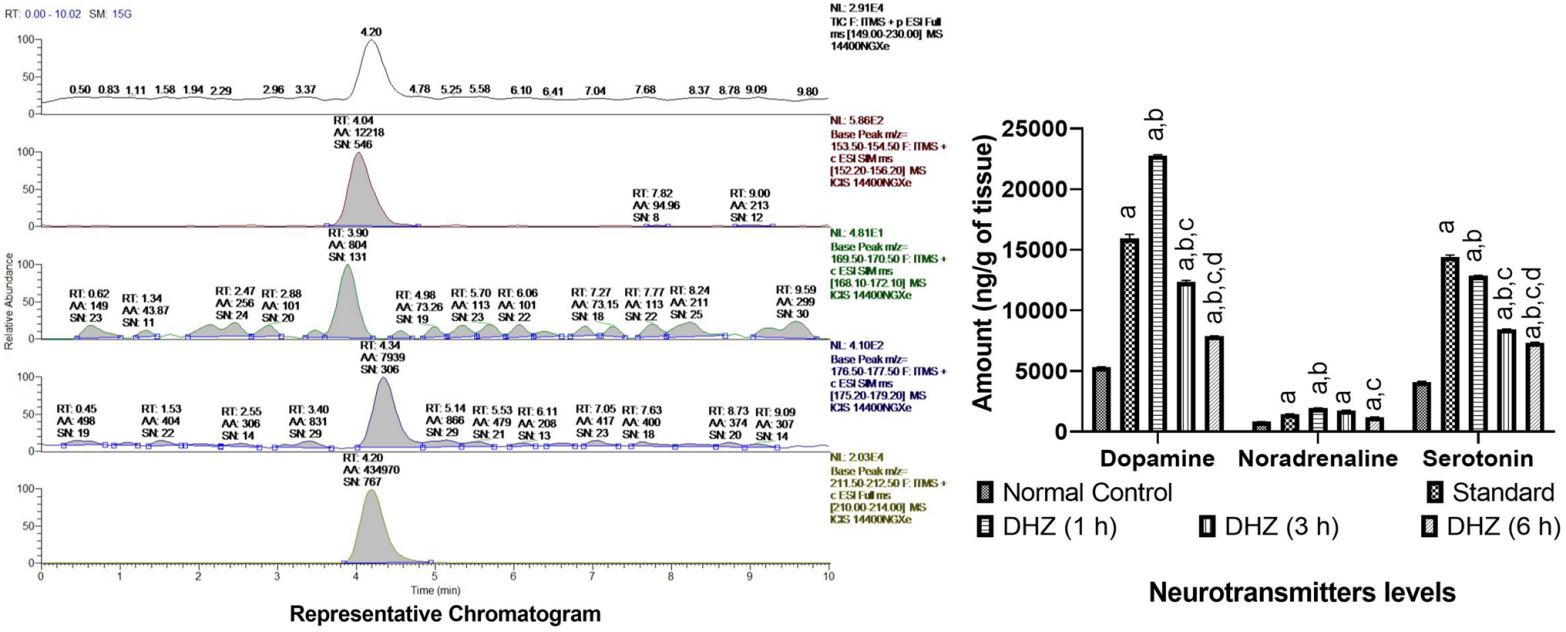
Neurotransmitter levels in whole brain. Legend: Two-way ANOVA followed by Tukey’s multiple comparison test was used for statistical analysis. The data are presented as mean ± SEM of six animals where ^a^*p* < 0.05 compared to normal control, ^b^*p* < 0.05 compared to escitalopram, ^b^*p* < 0.05 compared to DHZ (1 h), ^c^*p* < 0.05 compared to DHZ (3 h), ^b^*p* < 0.05 compared to DHZ (6 h)

LC–MS method was used to measure the level of serotonin, -noradrenaline and dopamine in the mice brain. Statistically significant changes were seen for interaction (*F*_8,75_ = 1027 and *p* < 0.0001) and neurotransmitters (*F*_2,75_ = 14,671 and *p* < 0.0001) within groups (*F*_4,75_ = 3514 and *p* < 0.0001). Normal control animals showed 5310.4 ± 31.48, 817.3 ± 6.58 and 4103.5 ± 43.43 ng/g of tissue, respectively, for dopamine, noradrenaline and serotonin levels in brain homogenate. Escitalopram treatment showed a significant increase in dopamine (*p* < 0.0001), noradrenaline (*p* = 0.0008) and serotonin (*p* < 0.0001) levels as compared to normal control. An increase in the level of these neurotransmitters was observed after treatment with DHZ as compared to normal control (Fig. 8). The significantly highest increase was found in the mice 1 h post-treatment for dopamine (*p* < 0.0001), noradrenaline (*p* < 0.0001) and serotonin (*p* < 0.0001) followed by 3 h post-treatment (*p* < 0.0001 for all three neurotransmitters) and 6 h post-treatment (*p* < 0.0001, *p* = 0.1214 and *p* < 0.0001 respectively) of DHZ s compared to normal control. The level of dopamine demonstrated as four times increase in comparison to the normal control group while serotonin levels were increased by nearly three times. Observed neurotransmitter levels are represented as bar diagrams and LC–MS chromatogram in Fig. 8

## Discussion

Ginger has been proven to possess antibacterial, antidiabetic, antiobesity activity, anti-inflammatory, anticancer activity, hepatoprotective and neuroprotective activity [38]. Zingerone, one of its active constituent, has been proven to possess an anti-inflammatory property by increasing the peroxisome proliferator-activated receptor (PPARs) [39]. The constituents of ginger, namely 6-gingerol and 6-shogaol, have been experimentally proven to modulate signalling molecules like STAT3, MAPK, PI3K, Bcl-2, caspases and possess chemotherapeutic activity in both in vitro and in vivo models [40]. Dehydrozingerone and series of its analogues were evaluated for their antioxidative, antityrosinase [9]; cytotoxic and antimicrobial activity, [15, 16] Isolation of DHZ can be done from the dried powdered rhizomes of ginger using hot methanol followed by chloroform partition as suggested by Kuo et al. [9]. This study provides insight into the involvement of MAO-A enzyme in catalysing the deamination of the neurotransmitters (chiefly dopamine, serotonin and noradrenaline) in the antidepressant activity of DHZ, supported by the shreds of evidence from in silico simulation, pharmacological and biochemical studies. Dysregulation of the neurotransmitters like dopamine, serotonin and noradrenaline in CNS has been proven to play an important role in the pathogenesis of depression [6, 21, 41]. Currently, most of the prescribed and efficacious antidepressants are serotonin or noradrenaline reuptake and/or monoamine oxidase inhibitors which act by increasing the synaptic availability of these neurotransmitters.

Martinez et al., have shown that the antidepressant activity of DHZ is due to the mechanism involving serotonergic and noradrenergic systems [22]. In this study, we have shown the involvement of the MAO enzyme system through the computational simulation-based study as well as by estimation of the brain level of neurotransmitters. We observed that the antidepressant activity of DHZ is likely due to inhibition of MAO enzyme that resulted in the increase in the level of the neurotransmitters dopamine, serotonin and noradrenaline in the brain of the mice. LC–MS based analysis of the mice brain homogenate also has shown a 3–fourfold increase in neurotransmitters in the DHZ treated group of mice as compared to the normal group. This increase in the level of neurotransmitters might be due to the inhibition of MAO-A enzyme as predicted by the computational simulation study.

Computational simulation-based docking studies have become a part of the drug discovery process involving hit identification to lead optimization [42]. It also helps understand the biological significance of ligand–protein interactions, the enzymatic mechanism using quantum mechanics [43]. In the present study, we have shown for the first time, the strong binding affinity between Dehydrozingerone and MAO-A enzyme as well as its stability using MMGBSA and 30 ns MD simulation study. Physicochemical property analysis with the Qikprop tool has predicted the possibility of DHZ to cross BBB and have good oral absorption with accepted Lipinski rule. This simulation-based study adds up the evidence that the antidepressant activity of the DHZ molecule is based on the inhibition of the MAO enzyme [44].

FST and TST are the widely used models for the evaluation of antidepressant activity [30, 45]. In the present study, for the confirmation of the antidepressant activity of DHZ, locomotor activity was also evaluated before FST to avoid false-positive results. The immobility time was significantly reduced in mice with post 1 h DHZ (100 mg/kg *po*) treatment and Escitalopram (10 mg/kg *po*) (selective serotonin reuptake inhibitor) treatment on the first day of the test as compared to the normal mice, while 6 h DHZ treatment showed no reduction in immobility time. On the 7th day of the test, further reduction in the immobility time was observed in the group with post 1 h and 3 h treatment of DHZ. This suggests the increased potential for the antidepressant activity of DHZ in the chronic treatment model. In addition, TST was also performed to ensure the antidepressant activity. A similar pattern of the decrease in the immobility time in TST was observed after DHZ treatment. Open field test was done to eliminate doubts regarding the reduction in immobility time in FST and TST, which might be due to the alterations of locomotor activity by the study drug. No change in the locomotor activity was observed by the dose of DHZ, which was used in the study for antidepressant activity.

Most of the current medications for depressions have less success rate of approximately about 60% with no response in some patients [46]. Current antidepressants have short-term benefits and take a long period to show effect. Most of these synthetic molecules show side effects on prolonged use and less significant difference is observed between the effect of active drugs and placebo [47]. There is a need for rapid-acting antidepressants with fewer side effects [48]. Plant-based molecules might have the potential to overcome these shortcomings. DHZ might be one such potential candidate with the expected antidepressant activity.

## Conclusion

The present study showed a similar antidepressant effect of DHZ (100 mg/kg *po*) to that of Escitalopram (10 mg/kg *po*) after 7 days treatments in acute depression-like studies namely FST, TST and OFT. The antidepressant effect of DHZ was more prominent at 1 h after treatment as compared to 3 h and 6 h of the treatment. The DHZ treatment elevated the neurotransmitters serotonin, dopamine and noradrenaline levels in the mice brain, which was in corroboration with its observed antidepressant activity. Computational-based virtual molecular docking and molecular dynamic simulation studies were performed to access the binding interaction of DHZ with MAO-A enzyme. DHZ showed good stable interaction and binding with the MAO-A enzyme. In silico molecular simulation study is predicting and pointing on the MAO-A inhibitory potential of DHZ as the molecular basis for the observed antidepressant activity. However, further experimental biological enzyme inhibiting potential of DHZ has to be further evaluated for confirmation of the MAO-A inhibiting potential of DHZ. In conclusion, DHZ, a half analogue of curcumin, has been proven to possess antidepressant activity in the in silico and in vivo study mediated through increased neurotransmitter level. However, further studies are required for confirmatory studies of MAO-A inhibitory activity at the molecular level.
